# Polygonatum polysaccharides as gut microbiota modulators: implications for autophagy-dependent PD-L1 clearance in cancer immunotherapy

**DOI:** 10.3389/fnut.2025.1612644

**Published:** 2025-06-24

**Authors:** Yongjie Li, Feng Jiang, Ting Wang, Min Zeng

**Affiliations:** ^1^School of Pharmacy, Shaoyang University, Shaoyang, Hunan, China; ^2^Southwest Hunan Research Center of Engineering for Development and Utilization of Traditional Chinese Medicine, Shaoyang, Hunan, China; ^3^Department of Nutrition, Taizhou Central Hospital, Taizhou, Zhejiang, China; ^4^The Second Affiliated Hospital, Hengyang Medical School, University of South China, Hengyang, Hunan, China; ^5^The Affiliated Shaoyang Hospital, Hengyang Medical School, University of South China, Shaoyang, Hunan, China

**Keywords:** gut microbiota, Polygonatum polysaccharides, autophagy, lysosome, PD-L1

## Abstract

The purpose of this review article is to examine the biotransformation of Polygonatum polysaccharides and the role that gut microbiota plays in that biotransformation. In the past years, PD-L1 has come under much attention as an immune checkpoint target and autophagy-lysosomal mediated degradation of PD-L1 provides new approaches for cancer immunotherapy. This review explores how gut microbiota-mediated biotransformation of Polygonatum polysaccharides may influence PD-L1 expression and degradation, potentially offering a novel approach to enhancing cancer immunotherapy. This synthesis of information highlights the complex relationship between foods, microbiota, and the immune system and supports the emerging idea that targeting microbiota-mediated metabolism and the immune response may be beneficial to improve cancer therapeutics.

## 1 Introduction

The gut microbiome is crucial for host health and plays a significant role in cancer enhancement through the modulation of disease processes. The gut microbiota has recently been implicated in immune response and therapeutic outcome in cancer treatments, especially in immunotherapy (1–3). Examining gut microbiota mechanisms and host immune function also contributes to our ability to develop enhanced therapeutic strategies to combat tumor growth (4–6). This review will detail the biotransformation of polysaccharides from Polygonatum and their capacity to modulate PD-L1 degradation via autophagy-lysosomal mechanisms and the gut microbiome’s role in this transformation.

The gut microbiome contains trillions of different microorganisms (bacteria, fungi and viruses) in the gastrointestinal tract, that are required for normal digestion and absorption of nutrients as well providing health benefits through immune response modulation, inflammation, and overall health. Alterations of the microbial communities, or dysbiosis, have been identified as contributors to numerous disease states, including cancer. Specific bacterial populations have been identified as contributors to the reduced effectiveness of immunotherapy by influencing the tumor microenvironment and impacting immune cell function (7, 8). Understanding the interaction of gut microbiota on cancer therapeutics known to promote immune exhaustion will help orchestrate more effective treatment strategies.

More recently, focus has shifted to the gut microbiome’s contribution to cancer immunotherapy outcomes through immune checkpoint inhibitors, PD-1 and PD-L1. PD-L1 is a protein expressed on tumor cells that interacts and binds with PD-1 on T cells, effectively inhibiting T cell activity and immuno-editing to allow tumor clearance. Recent studies have also determined that gut microbiota influence PD-L1 expression and degradation by modulating the immune response to tumors through alterations in immune responses (9–12). Specific bacteria in gut microbiota enhance the efficacy of PD-1 monoclonal antibodies in anti-cancer treatment by modulating the immune landscape (13, 14). This suggests that knowledge of gut microbiota composition could impact treatment response.

Polygonatum polysaccharides may demonstrate physiological responses benefiting human health and enhancing the immune system with anti-inflammatory and immunomodulatory properties. Studies have also shown that polysaccharides from Polygonatum may modulate gut microbiota composition, while enhancing the production of beneficial short-chain fatty acids (SCFAs) from gut microbiota (15–17). The bioactive characteristics of polysaccharides allow them to be resistant against digestion, with the ability to pass through the tract to reach the colon intact for fermentation by gut microbiota. The fermentation process will induce changes in microbiota activities and diversity toward SCFAs production that can help maintain gut barrier integrity and modulate immune system responses (18).

Furthermore, the autophagy-lysosomal mechanism has been recognized as a key mechanism for driving the degradation of PD-L1. Autophagy is a cellular mechanism that degrades and recycles damaged organelles and proteins, such as PD-L1. Recent studies have shown that activating autophagy can drive increased degradation of PD-L1, restore T cell functionality, and enhance the anti-tumor immune response (19, 20). Together, these interactions provide a novel therapeutic mechanism for the enhancement of cancer immunotherapy utilizing both gut microbiota and Polygonatum polysaccharides.

In summary, the gut microbiome has a complicated capacity to benefit host health in cancer immunotherapy. The transformation of Polygonatum polysaccharides in the gut microbiota can enhance PD-L1 degradation through the autophagy-lysosomal pathways, thereby facilitating immune responses to tumors. Future studies should focus on determining the microbial mechanisms associated with PD-L1 degradation and the mechanism by which Polygonatum polysaccharides impact such outcomes. Understanding these mechanisms is critical for developing innovative membrane theories and improving immunotherapy, which can have positive implications for patient outcomes.

## 2 Overview of the gut microbiome

The gut microbiome, a diverse collection of microorganisms inhabiting the human gastrointestinal tract, has been shown to be an important element in human health and disease. The gut microbiome contains trillions of bacteria, archaea, viruses and fungi that contribute to the regulation of numerous important biological processes such as digestion, metabolism, and immunity. Recent advances in sequencing technologies have allowed researchers better characterization of the gut microbiome and its interaction with the human host. These gut microbiota can be selectively modulated by a variety of factors including diet, environment, lifestyle and genetics resulting in significant variation in the microbiome of different individuals. Most importantly, the gut microbiome is not a passive inhabitant of the gut; rather, gut microbes engage in direct interactions with the host metabolism by producing vitamins, SCFAs and other metabolites required for homeostasis (21, 22). Knowledge of the microbiota composition and function is critical to understanding the role of the microbiome in human health and disease, especially as it pertains to metabolic disorders, auto-immune disease and infection.

### 2.1 Composition and function of the gut microbiome

The gastrointestinal microbiome consists of a highly diverse community of microbes, primarily bacteria, that fall into multiple phyla, which in turn contain numerous genera and species (namely Firmicutes, Bacteroidetes, Actinobacteria, Proteobacteria). The microbial diversity among individuals composing the gut microbiome (or gut microbiome profile) can vary based on age, diet, antibiotic use, and environmental exposures (23, 24).

The gut microbiome serves more than one metabolic function, such as digesting carbohydrates that cannot be digested by human enzymes and producing SCFAs, including butyrate, propionate, and acetate, to provide energy to colonocytes and regulate inflammatory and immune responses (25, 26). The gut microbiome is involved in the production of key vitamins, such as vitamin K and the B group of vitamins, and has a role in bile acid metabolism critical for fat digestion and absorption (27, 28).

In addition to metabolic functions, the gut microbiome is also considered to be a major part of the immune system. It helps to educate and modulate the immune response by developing tolerance to commensal organisms while maintaining the capacity to respond to pathogens. Disturbances in the gut microbiome composition (or dysbiosis) have been implicated in several ailments, including inflammatory bowel disease, obesity, diabetes, and mental disorders (24, 29). These implications highlight the importance of the gut microbiome on the health, wellness, and wellbeing of the individual.

### 2.2 Interaction between the microbiome and host metabolism

The relationship between the gut microbiome and host metabolism is intricate and dynamic, and importantly impactful on health. The gut microbiome produces microbial metabolites, including SCFAs, to impact host metabolic pathways. An example is butyrate, a SCFA mainly produced by the fermentation of dietary fibers by gut bacteria, which can improve host insulin sensitivity, lipid metabolism, and decrease inflammation (27, 29, 30). The gut microbiome also influences target drugs and other nutrients through metabolism leading to changes in the bioavailability and biological effects of the nutrient. There are gut bacteria that can metabolize plant-based dietary polyphenols into beneficial bioactive metabolites affecting host metabolism (31–33).

The gut microbiome can also influence energy balance in the host by modulating appetite and energy metabolism and specific gut microbiome compositions have been associated with obesity and metabolic syndrome (27, 28, 34, 35). An additional example of how the gut microbiome can impact host metabolism is through a gut–brain axis, which is a bidirectional signaling pathway between the gut and the central nervous system. The gut–brain axis has been implicated in behaviors, and consequently host metabolic pathways linked to behaviors such as anxiety or depression (29, 36).

Understanding the interrelationship of these factors is essential for developing interventions, such as probiotics, prebiotics, and dietary modifications, to improve intestinal microbiome composition and its health-beneficial functions (37–39).

### 2.3 Role of the microbiome in immune regulation

The gut microbiome is fundamentally important for regulating immune system function, serving as a barrier to pathogens and eliciting immune tolerance to commensal organisms. This immunomodulatory role is necessary to maintain homeostasis and avoid excessive inflammatory responses that could predispose individuals to autoimmune diseases (40, 41).

Microbial-derived metabolites, such as SCFAs, are crucial in shaping immune responses. They influence the differentiation and function of various immune cells, including regulatory T cells (Tregs), which are essential for maintaining immune tolerance (27, 29, 42). The gut microbiome may also curtail the production of cytokines and other signals associated with inflammation and immune processes (28, 36).

Dysbiosis, characterized by a reduction in microbial diversity and an imbalance in microbial composition, has been associated with various immune-mediated conditions, including inflammatory bowel disease, allergies, and even cancer (21, 22). This suggests that a balanced gut microbiome likely benefits optimal immune system function.

Recent studies have even identified potential to utilize modulation of the microbiome as a strategy for enhancing immune response in the context of cancer immunotherapy. The gut microbiome is known to influence the effectiveness of immune checkpoint inhibitors; thus, interventions to restore a healthier microbiome may enhance treatment (43, 44).

**Figure 1:**
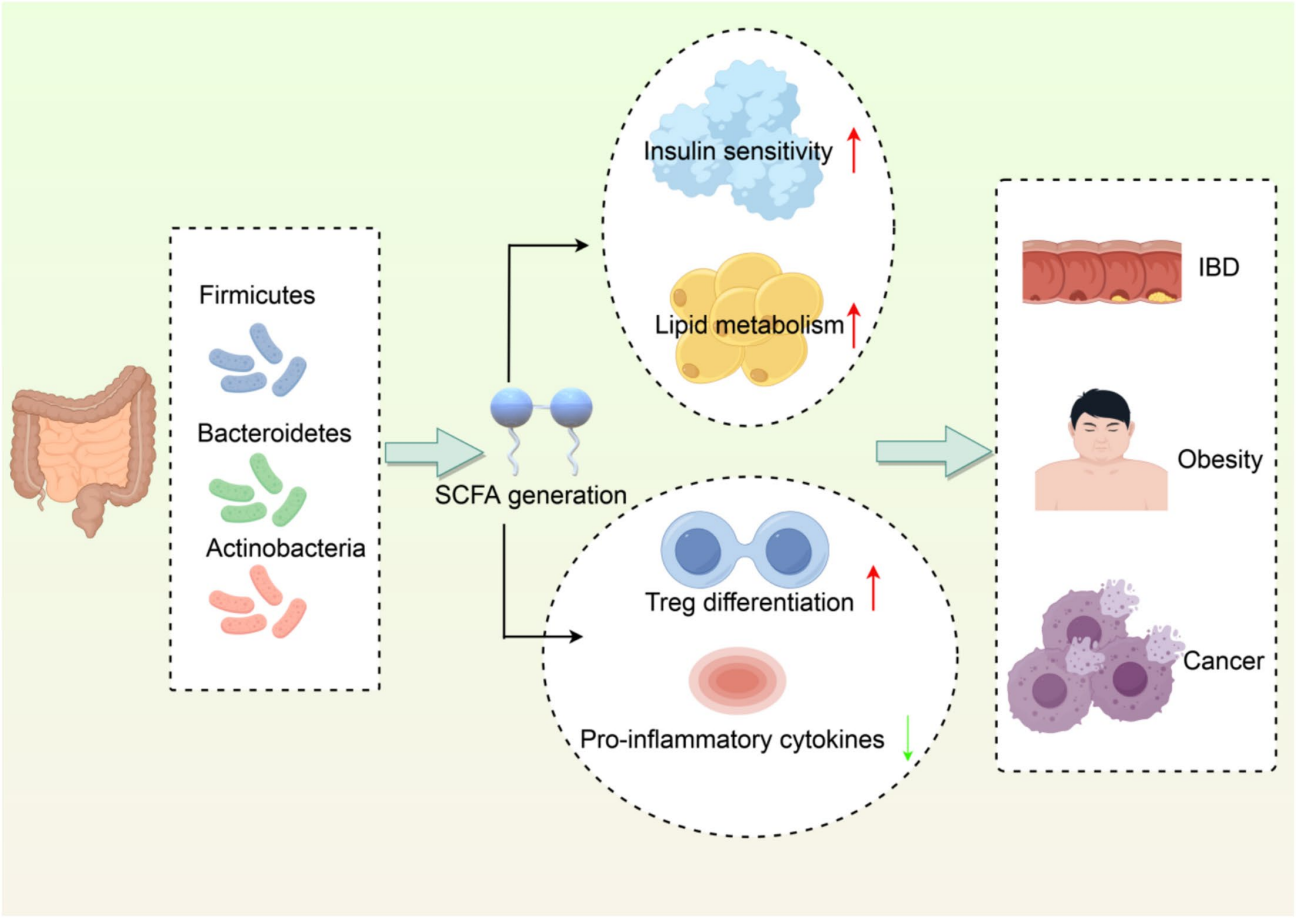
Interaction network between gut microbiota and host metabolism/immune regulation. (1) Dominant phyla (Firmicutes, Bacteroidetes, Actinobacteria) producing SCFAs. (2) SCFAs enhance insulin sensitivity and lipid metabolism. (3) SCFAs promote Treg differentiation and suppress inflammation. (4) Dysbiosis links to IBD, obesity, and cancer. Created with Figdraw.com.

In conclusion, the gut microbiome is a critical component of immune regulation and can impact host health and disease processes in interaction with the immune system. Studying these interactions will be valuable in guiding the development of novel therapeutic strategies based on using the microbiome to improve health outcomes (Figure 1).

## 3 Characteristics of Polygonatum polysaccharides

### 3.1 Sources and extraction of Polygonatum polysaccharides

Polysaccharides derived from Polygonatum species, including *Polygonatum cyrtonema*, *Polygonatum sibiricum*, and *Polygonatum kingianum* are often used as herbal medicine in Traditional Chinese Medicine due to their medicinal properties. Polygonatum polysaccharides are typically extracted using a variety of methods, including hot water extraction, ethanol precipitation, or an alternative approaches that include modern chromatographic techniques like Diethylaminoethyl (DEAE) cellulose column chromatography or gel filtration. In studies with *Polygonatum cyrtonema*, polysaccharides can be effectively extracted by hot water extraction combined with purification procedures and analysis of bioactive properties of polysaccharides have been reported (45). Additionally, extraction procedures for polysaccharides can physically and chemically alter their properties and the analysis of the extracting parameters can have a dramatic impact on the outcome of isolation, where some of the key extracting variables can be temperature, time, or solvent (46). Extraction conditions can also play a role in molecular weight and monosaccharide composition and bulk extraction conditions can impact the biological activity of polysaccharides (15, 47). Therefore knowledge of extraction conditions is important when trying to assess the therapeutic potential of Polygonatum polysaccharides (Table 1).

**Table 1:** Physicochemical properties and biological activities of Polygonatum polysaccharides from different sources.

Source species	Extraction method	Molecular weight (kDa)	Main monosaccharide composition	Biological activities	References
*Polygonatum cyrtonema*	Hot water extraction + DEAE purification	120–150	Glucose, Fructose	Immunomodulation, Anti-inflammatory	(45)
*Polygonatum sibiricum*	Ethanol precipitation	80–100	Mannose, Galactose	Antioxidant, Gut barrier protection	(50, 51)
*Polygonatum kingianum*	Ultrasound-assisted extraction	200–300	Arabinose, Xylose	Microbiota modulation, SCFA production	(18)

### 3.2 Biological activity of Polygonatum polysaccharides

Polygonatum polysaccharides exhibit a wide range of biological activities and are recognized as potential health-promoting food and possible medicinal products. Scientific investigations have demonstrated that Polygonatum polysaccharides possess immunoregulatory, anti-inflammatory, antioxidant, and prebiotic activities. For example, the polysaccharides from *Polygonatum sibiricum* stimulated an immune response by increasing the production of specific cytokines and promoting inflammation, which contributes to health and disease resilience (48–51). In addition to their effect on the immune response, Polygonatum polysaccharides are antioxidants and limit oxidative stress and inflammation (52). As such, Polygonatum polysaccharides may also be effective treatments for ulcerative colitis and other inflammatory disorders. Furthermore, recent evidence demonstrates the ability of polysaccharides to regulate gut microbiota composition, through the promotion of beneficial bacteria (e.g., Bifidobacterium and Lactobacillus) and the reduction of pathogenic bacteria (53). Prebiotic activity is critical to gut health and maintaining diversity in human microbiota in mitigating dysbiosis associated with various metabolic disorders. Lastly, Polygonatum polysaccharides can also influence the regulation of SCFA concentrations. SCFAs are important in maintaining gut health as they contribute to intestinal barrier integrity and overall metabolic health (18). In addition to their roles in gut health, Polygonatum polysaccharides exhibit anticancer effects, particularly in cell lines such as MCF-7, HepG2, and A549. They induce apoptosis and inhibit cell proliferation, with studies demonstrating that they can cause G1 phase arrest and promote apoptosis in HepG2 cells through intrinsic pathways. Notably, the efficacy of these polysaccharides can be enhanced with higher doses and prolonged exposure (54). Variations in their structural characteristics, such as molecular weight and glycosylation, significantly impact their anticancer activities, with some polysaccharides directly inducing cancer cell death, while others enhance immune responses or inhibit tumor growth through indirect mechanisms (55). In summary, the biological activities of Polygonatum polysaccharides support the use of these polysaccharides and their extracts as functional food ingredients in food and medicine.

### 3.3 Potential as natural medicines

Polysaccharides from the Polygonatum genus are gaining interest as natural medicines due to their diverse pharmacological effects and low toxicity. Polygonatum polysaccharides have shown to provide protection from a variety of diseases including metabolic disease, neurodegenerative disease, and gastrointestinal disorders, while also exhibiting low toxicity. A few studies investigated the effect of Polygonatum polysaccharides on glucose and lipid metabolism in animal models of diabetes, providing reasonable evidence for their use in diabetes or diabetes-related complications (56). In addition to metabolic effects, Polygonatum polysaccharides may also enhance cognition in cognitive impairment models, indicating their potential for neuroprotective properties (57, 58). Finally, the anti-fatigue potential of Polygonatum polysaccharides is demonstrated in animal studies, where plausibly suggesting their role in relate to performance and recovery (59). Furthermore their ability to regulate the gut-brain axis in treatment of mental illness is a novel area or research, where Polygonatum polysaccharides have shown behavior changes related to modulation of gut microbiota (60). Overall, the therapeutic potential of Polygonatum polysaccharides suggests the potential to develop natural medicines, functional food products, and dietary supplements that promote health and prevent disease.

### 3.4 Polysaccharides as autophagy modulators: beyond Polygonatum

The immunomodulatory effects of polysaccharides via autophagy-lysosomal pathways are not unique to Polygonatum species. A growing body of evidence suggests that multiple polysaccharides and oligosaccharides from diverse sources share the ability to regulate autophagy and immune checkpoints like PD-L1. For example: d-Mannose, a monosaccharide derivative, has been shown to trigger TFE3-mediated lysosomal degradation of PD-1/PD-L1, highlighting the conserved role of carbohydrate metabolites in immune checkpoint regulation (61).

## 4 The role of PD-L1 and targeted therapy

### 4.1 Biological functions of PD-L1

PD-L1 serves as a significant immune checkpoint molecule that is critical in regulating immune responses particularly in the context of malignancy. PD-L1 is a protein expressed on a variety of immune cells such as dendritic cells, macrophages, and T-cells, and also on tumor cells. PD-L1’s primary role is to engage PD-1 receptor on activated T-cells leading to T-cell inhibition, reduced cellular activation and reduced proliferation. As a result of engagement between PD-1 and PD-L1, immune responses are inhibited, allowing tumors to evade immune detection while also promoting immune enhancement for tumor progression (62).

In addition to being an immune checkpoint regulator, PD-L1 has intrinsic functions that additionally remain important in the tumor biology narrative. Studies have shown PD-L1 is capable of modulating cellular behaviors related to proliferation, survival, and migration independent of PD-1 (63, 64).

PD-L1 expression is modulated by external factors including immune and pro-inflammatory cytokines, as well as signal transduction pathways that become activated in the tumor microenvironment. For example, IL-6 can initiate the JAK/STAT pathway which enhances PD-L1 expression while also having downstream consequences for immune responses impacted by the tumor (65). The descriptive roles played by PD-L1 in cancer and its characterization will provide opportunities to develop new immunotherapies that change the roles of PD-L1 as an immune checkpoint modulator and its intrinsic tumor supporting functions.

### 4.2 The role of PD-L1 in the tumor microenvironment

The tumor microenvironment (TME) is a complicated and ever-changing ecosystem of tumor cells, immune cells, stromal cells, and extracellular matrices. PD-L1 is a key player in the TME and has an effect on tumor growth and the immune response. When expressed in tumors, PD-L1 expression is associated with a worse prognosis, as it supports tumor progression and immune evasion (66).

When in the TME, PD-L1 can interact with various immune cells, including T cells, natural killer (NK) cells, and macrophages. Once bound to PD-1 on T cells, PD-L1 will inhibit T cell activation, proliferation, and production of cytokines, thus leading to T cell exhaustion (67). PD-L1-mediated immune suppression promotes tumor growth as the adaptive immune response can be abrogated. PD-L1 expression by macrophages can also bolster the immunosuppressive properties of this cell type, which also aids in promoting immune suppression in the TME (68).

**Figure 2:**
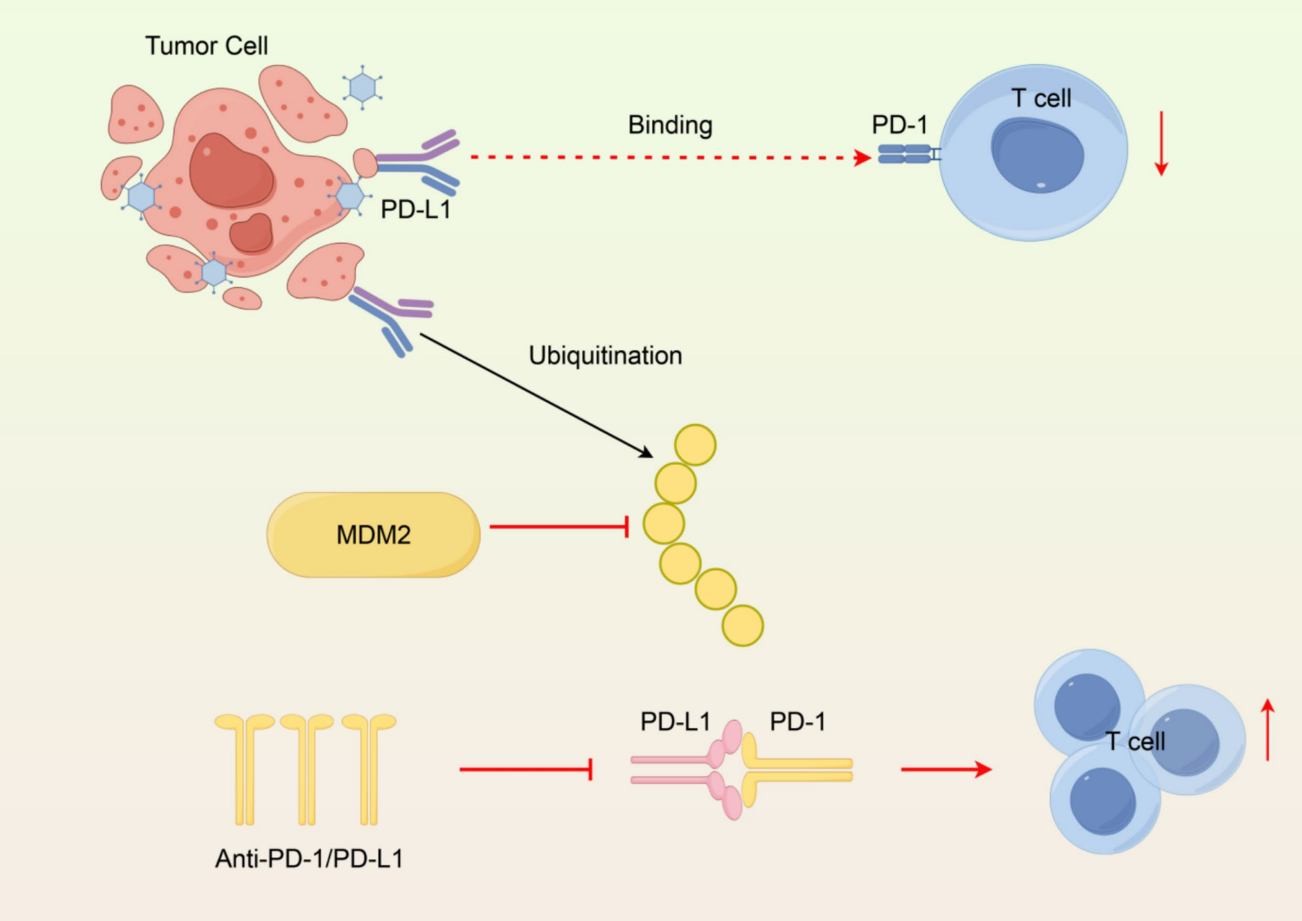
Immunosuppressive mechanism of PD-L1 in the tumor microenvironment. (1) PD-L1 binding PD-1 inhibits T cells. (2) MDM2 ubiquitinates PD-L1 for degradation. (3) Anti-PD-1/PD-L1 antibodies block interaction, restoring T cell activity. Created with Figdraw.com.

PD-L1 in the TME may also promote the recruitment of Tregs that further suppress immune responses against tumors (69). This creates a feedback loop in which PD-L1 diminishes effector T cell activity while also promoting Tregs accumulation and enhancing immunosuppression overall. The role of PD-L1 in TME dynamics will be important for the development of combination therapies that counteract PD-L1-mediated immunosuppression and increase anti-tumor immune responses (Figure 2).

### 4.3 Recent research progress on immune checkpoint inhibitors

The emergence of immune checkpoint inhibitors (ICIs) targeting the PD-1/PD-L1 axis has dramatically changed the landscape of cancer treatment and provided new treatment opportunity for a variety of cancers. ICIs prevent the interaction between PD-1 and PD-L1 to restore T-cell activity and boost anti-tumor immunity. Clinical trials have demonstrated that using anti-PD-1 and anti-PD-L1 antibodies is helpful in treating a variety of cancers: example, melanoma, lung cancer, and renal cell carcinoma (70).

There has also been an effort to understand the mechanisms of resistance to ICI therapy since not all patients will respond. Tumor mutational burden, PD-L1 levels, and composition of TME are strong indicators of responder status (71). Combination therapies with chemotherapy or radiation may enhance ICI efficacy (72–74).

Furthermore, new strategies for overcoming resistance mechanisms are being evaluated. For example, targeting the intracellular domain of PD-L1 is an emerging strategy that is intended to increase the efficacy of PD-1/PD-L1 blockade by destabilizing PD-L1 and promoting PD-L1 degradation (75, 76). Additionally, the use of nanoparticles for targeted delivery of PD-L1 inhibitors has been evaluated to promote specificity and lower the systemic toxicity of PD-L1 therapies (77, 78).

Overall, ongoing research into PD-L1 and the role of PD-L1 in cancer immune therapy will continue to be informative for the development of more effective treatment strategies will lead to improved patient outcomes in cancer biology.

## 5 Mechanism of the autophagy-lysosome pathway

### 5.1 Basic process of autophagy

Autophagy is a crucial cellular mechanism that is involved in maintaining cellular homeostasis via the degradation and recycling of damaged organelles, misfolded proteins, or intracellular pathogens. Autophagy is essential in cells experiencing stress conditions such as nutrient deprivation and oxidative stress. Autophagy is initiated by the formation of phagophores, which is double-membrane structure that engulfs the cellular organelles or proteins to be degraded. The phagophore elongates and subsequently closes to form the autophagosome. The autophagosome fuses with lysosomes, forming an autolysosome, where the targeted cargo is degraded by lysosomal enzymes. Degraded components of the cell are recycled, providing protective mechanisms against various pathological diseases, including cancers and neurodegenerative diseases (79). Collectively, autophagy is essential for cellular health and disease.

### 5.2 Lysosomal function and PD-L1 degradation

**Figure 3:**
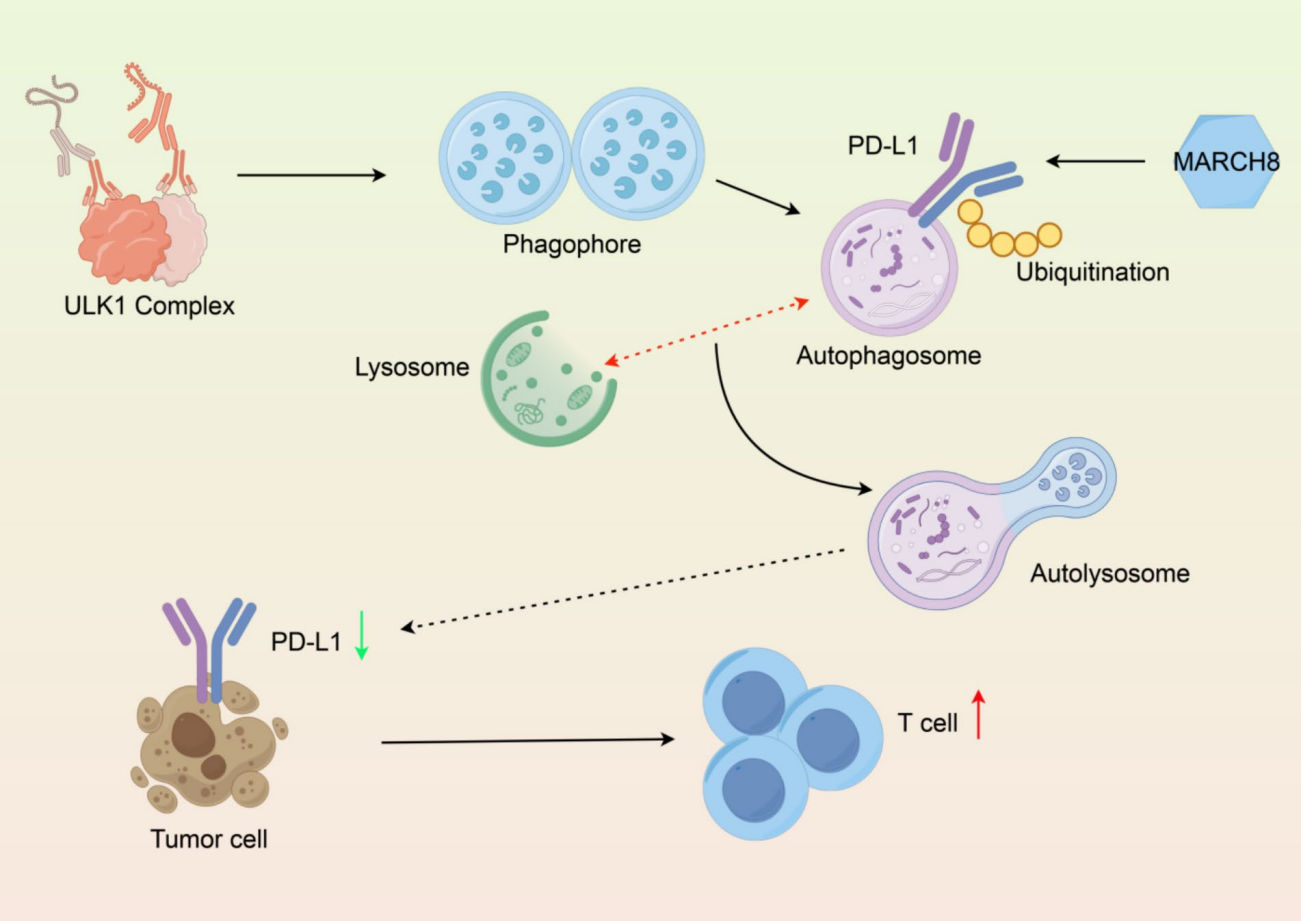
Molecular mechanism of PD-L1 degradation via the autophagy-lysosomal pathway. (1) Autophagosome formation, (2) Ubiquitinated PD-L1 encapsulation, (3) Lysosomal fusion, (4) Functional output of PD-L1 degradation and T cell activation. Created with Figdraw.com.

Lysosomes are cellular compartments that are bound by a membrane that contain hydrolytic enzymes that degrade biomolecules. Lysosomal degradation is a key part of the autophagy-lysosome pathway and mediates the degradation of PD-L1 protein, which is an important immune checkpoint protein expressed on the surface of tumors. Tumor cells frequently overexpress PD-L1, allowing for the immunoediting of cancer cells by binding PD-L1 on tumor surfaces to PD-1 on T cells, thus inhibiting their anti-tumor activity. Recent studies have demonstrated that lysosomal degradation of PD-L1 can enhance anti-tumor immunity by decreasing the abundance of PD-L1 on the surface of tumor cells, while also stimulating T cell activation (80). The lysosomal degradation of PD-L1 can occur through multiple mechanisms including ubiquitination and selective E3 ligases, such as MARCH8, which targets PD-L1 for lysosomal degradation. Understanding the mechanisms of PD-L1 degradation is essential for developing therapeutic strategies to enhance anti-tumor immunity and improve the effectiveness of immune checkpoint inhibitors (Figure 3) (81).

### 5.3 Relationship between autophagy and gut microbiota

The relationship between autophagy and the gut microbiota is a burgeoning area of research that highlights the intricate interplay between host cellular processes and microbial communities. Autophagy plays an essential role in the maintenance of gut homeostasis due to the fact that it plays a role in maintaining the composition and physiological activity of the gut microbiota. Autophagic dysregulation may alter microbial diversity and composition which can impact the host health and susceptibility to disease. For example, prior studies indicate that autophagy-related genes are required for removing pathogenic bacteria and for the maintenance of beneficial microbes in the gut (82, 83). Moreover, the gut microbiota may also influence autophagy, indicating that microbial metabolites may modulate autophagy and vice versa. This relationship may be important in the context of specific diseases characterized by dysregulated autophagic activity and gut microbiota, such as inflammatory bowel diseases (IBD) and metabolic disorders (84, 85). Understanding the role of autophagy in conjunction with the gut microbiota could contribute to the development of novel strategies targeted toward both populations to benefit overall health.

### 5.4 Autophagy-lysosomal pathway as a PD-L1 degradation hub

The autophagy-lysosomal pathway has emerged as a critical regulator of PD-L1 protein homeostasis. Emerging evidence indicates that PD-L1 can be selectively degraded through ubiquitination-dependent targeting to lysosomes. For instance, the E3 ubiquitin ligase MARCH8 mediates PD-L1 ubiquitination at lysine residues, promoting its recognition by autophagy adaptors (e.g., p62/SQSTM1) and subsequent sequestration into autophagosomes for lysosomal degradation (86). Similarly, small molecules such as SA-49 have been shown to enhance PD-L1 degradation by activating the lysosomal biogenesis regulator MITF, which coordinates the fusion of autophagosomes with lysosomes (87). These studies collectively highlight the lysosome as a central hub for PD-L1 clearance.

### 5.5 Bridging autophagy and biotransformation: metabolite-driven PD-L1 clearance

Microbial biotransformation of Polygonatum polysaccharides generates SCFAs that bridge gut microbiota activity to autophagy-dependent PD-L1 clearance. Butyrate inhibits histone deacetylase (HDAC) to upregulate autophagy genes (e.g., ATG5), while acetate activates GPR43-AMPK signaling to suppress mTORC1 and trigger autophagosome formation via ULK1. Concurrently, SCFA-induced reactive oxygen species (ROS) activate NRF2-mediated pathways, amplifying autophagic flux. These metabolite-driven mechanisms—epigenetic, receptor, and redox signaling—collectively link microbial metabolism to PD-L1 degradation, enhancing antitumor immunity.

## 6 Polygonatum polysaccharides: biotransformation and regulatory mechanisms

### 6.1 Effects of Polygonatum polysaccharides on gut microbiota

Polygonatum polysaccharides, specifically those isolated from species such as *Polygonatum cyrtonema* or *Polygonatum sibiricum*, have raised considerable interest in the context of the microbiome in human health. Specifically, these polysaccharides are found to resist digestion and arrive intact in the colon to be fermented by microbiome bacteria. Studies have noted that a homogeneous polysaccharide fraction of *Polygonatum cyrtonema* was confirmed to be an agavin-type fructan that resisted digestion and was found to promote additional SCFAs production during *in vitro* fermentation, in conjunction with decreasing pH, increasing beneficial genera (e.g., Bifidobacterium and Megamonas), and decreasing pathogenic genera (e.g., Escherichia-Shigella and Klebsiella) (88, 89).

Additionally, studies on various Polygonatum species have shown the capacity of polysaccharides to enrich gut bacterial diversity and richness, e.g., polysaccharides isolated from *Polygonatum kingianum* selected for beneficial probiotic genera while simultaneously restricting potentially detrimental genera, indicating the opportunity for as functional foods to reduce illness and improve gut health (18). In conjunction with the evidence showing the ability to promote SCFAs-producing bacteria, it is also an important means of sustaining gut health and preventing barriers to health relating to metabolic disorders (90, 91).

The interaction between Polygonatum polysaccharides and gut microbiota suggests the potential therapeutic targets for gut health and metabolic disorders. This physiological process of enhanced growth of beneficial microorganisms and restriction of pathogenic bacteria potentially leads to the replacement of disrupted microbial communities that could be associated with illness such as obesity, diabetes, and inflammatory bowel diseases (92). This gut microbiota regulatory process serves to promote gut health and could also carry relevance for systemic health, e.g., cognition and immune health, all of which imbues synergies of other health-conscious food products to manage and/or enhance gut health with Polygonatum polysaccharides (15).

### 6.2 Biotransformation products and their biological activities

The biotransformation process of polysaccharides derived from Polygonatum produces numerous health-promoting and biologically active compounds. During polysaccharide fermentation by gut microorganisms, SCFAs such as acetate, propionate, and butyrate are produced through their degradation, which has been shown to have anti-inflammatory effects while being protective of gut epithelium (93, 94). These SCFAs play a pivotal role in maintaining gut barrier integrity, regulating immune responses, and influencing metabolic pathways, thus contributing to the overall health of the host.

Evident data from studies has shown that polysaccharides derived from Polygonatum species will remit intestinal permeability and serum endotoxin levels, which are two components associated with the development of inflammatory and metabolic disorders (15). For example, polysaccharides from *Polygonatum sibiricum* improved various symptoms of colitis by modifying inflammatory cytokines and restoring the gut microbiome (92). This supports further evidence of polysaccharide biotransformation products to be therapeutic to gastrointestinal disorders.

Moreover, polysaccharides derived from *Polygonatum kingianum* can alter bioactive compounds depending on their sugar composition and molecular weight. For instance, polysaccharides from this species, which exist in various fractions resulting in altered prebiotics, exhibited different effects by promoting bactericidal and pathogenic strains (18). These results suggest potential in the purification and fractionation of polysaccharides from Polygonatum, indicating mechanistic pathways for functional foods and/or supplements to prevent disturbances in gut health and promote overall gut health.

Biotransformation products of Polygonatum polysaccharides will also exhibit influence with neuroprotective effects. Studies have beginning to suggest that Polygonatum polysaccharides have impact on the gut-brain axis affecting cognitive functions or mood (95, 96). This connection between gut health and neurological outcomes underscores the importance of dietary polysaccharides in promoting both physical and mental wellbeing.

### 6.3 Molecular mechanisms of autophagy-lysosomal pathway modulation

The relationship of Polygonatum polysaccharides with health status is a multifaceted relationship focusing on the potential regulation of the autophagy-lysosomal pathway. Autophagy is a core mechanism of cellular degradation and the recycling of defective organelles and proteins, and is paramount to cellular homeostasis. Emerging evidence suggests that Polygonatum polysaccharides modulate autophagy-related pathways, thus possibly acting as mediators of the theoretical health benefits attributed to Polygonatum polysaccharides.

As a case in point, polysaccharides extracted from *Polygonatum cyrtonema* have demonstrated the capability of ameliorating intestinal barrier function through the regulation of the expression of tight junction proteins, such as zonula occludens-1 (ZO-1) and occludin, that aid in the maintenance of the epithelial integrity of the colonic mucosa (92). This regulation occurs through autophagy modulation as degradation of damaged cellular components leads to a restoration of proper cellular function and reduced inflammation. Additionally, polysaccharides have also been shown to attenuate excessive inflammatory responses through modulation of pro-inflammatory cytokine secretion that is associated with autophagy modulation (97, 98).

Moreover, the association between Polygonatum polysaccharides and the gut microbiome may further modulate the role of autophagy. Polyssacharides can indirectly regulate autophagy in intestinal cells through the promotion of beneficial microorganisms and SCFAs production-which in turn can improve the intestinal barrier and reduce inflammation (99, 100). Notably, microbial metabolites derived from Polygonatum polysaccharides fermentation, particularly SCFAs, may serve as key mediators linking gut microbiota to autophagic activity. SCFAs (e.g., butyrate and acetate) regulate autophagy-related pathways through activation of G protein-coupled receptors (GPR41/43). Upon GPR41/43 activation, the downstream AMPK/mTOR signaling axis is triggered: AMPK phosphorylation inhibits mTORC1 activity, thereby relieving its suppression on the autophagy-initiating ULK1 complex. This promotes the assembly of the Beclin-1 and Atg5-Atg12-Atg16 complexes, as well as the conversion of LC3-I to LC3-II, ultimately driving autophagosome formation. This pathway may represent the core mechanism by which Polygonatum polysaccharides regulate PD-L1 degradation. Currently, no studies have conclusively demonstrated that Polygonatum polysaccharides directly promote PD-L1 degradation through autophagy. We propose that this hypothesis warrants experimental validation using genetic models (e.g., Atg5-deficient mice) and pharmacological inhibitors of autophagy. Current evidence is limited to: (1) PSP-induced elevation of the LC3-II/LC3-I ratio in colonic tissues (indirect evidence of autophagy activation) (15), and (2) SCFA-mediated lysosomal degradation of PD-L1 *in vitro* models (80). This proposal supports the premise that Polygonatum polysaccharides may be not only a prebiotic, but a modulator of fundamental cellular components of health.

**Figure 4:**
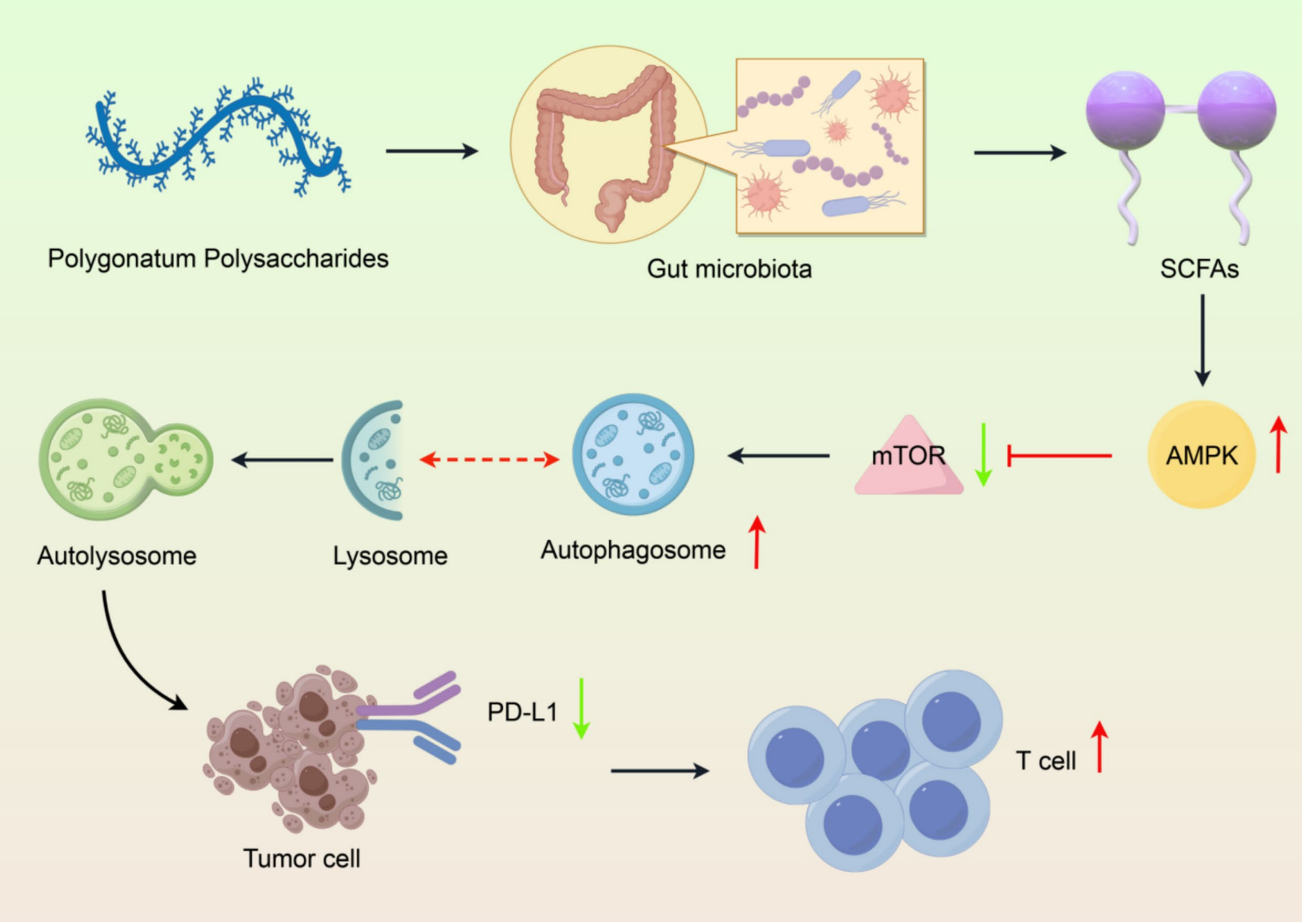
Integrated mechanism of Polygonatum polysaccharide-gut microbiota-autophagy axis in PD-L1 degradation. (1) Polysaccharide structure, (2) Gut microbiota modulation, (3) SCFA production, (4) Autophagy activation, (5) PD-L1 degradation and immune enhancement. Created with Figdraw.com.

In conclusion, the biotransformation of Polygonatum polysaccharides and their effects on gut microbiota, along with their modulation of the autophagy-lysosomal pathway, underscore their potential as functional food ingredients with significant health benefits. Critically, the hypothesis that Polygonatum polysaccharides promote PD-L1 degradation through autophagy requires validation via genetic knockout models (e.g., Atg5-deficient mice) or pharmacological autophagy inhibitors. Future studies on the specific molecular mechanisms and bioactive molecules generated from these polysaccharides will be needed to provide the basis for targeted therapeutic strategies to improve gut health and overall health (Figure 4).

## 7 Conclusion

The complex interactions between the gut microbiota and different therapeutic agents have received considerable attention in recent years, especially in responses to natural products such as Polygonatum polysaccharides. This review expounds the significant roles gut microbiota serve in the biotransformation of Polygonatum polysaccharides, and how this biotransformation may influence PD-L1 degradation in the autophagy-lysosome pathway. With this information, there is an academic and clinical basis for developing new strategies in the management of cancer patients with a focus on immunotherapy.

Key unresolved questions include: (1) Whether interindividual variability in gut microbiota composition affects the polysaccharide-autophagy-PD-L1 axis; (2) Long-term safety of microbial interventions (e.g., SCFA overproduction-induced intestinal barrier dysfunction); (3) Relationship between autophagy activation thresholds and tumor types; (4) Systematic analysis of polysaccharide structure–activity relationships (molecular weight, glycosidic bonds).

The gut microbiota functions as a highly adaptable ecosystem with the potential to alter the pharmacokinetics and pharmacodynamics of a range of compounds, including polysaccharides of natural origins. The results within this review highlight the need to gain a comprehensive understanding of the microbial communities that transform dietary polysaccharides into bioactive metabolites that may enhance or inhibit various biological pathways, including tumorigenesis and immune function. Connecting microbiome research and natural product pharmacology allows a greater understanding of countervailing interactions that impact therapeutic efficacy.

One of the most interesting insights from this review is its discussion of the autophagy-lysosome pathway as a possible way the gut microbiota alters PD-L1 degradation. PD-L1 is an important immune checkpoint protein that has emerged as a notable target for cancer therapy, especially with immune checkpoint inhibitors in mind. The gut microbiota’s ability to modulate PD-L1 levels via biotransformation pathways could serve as a new avenue of enhancing the efficacy of current immunotherapies. This linkage goes beyond expanding our knowledge on immune modulation and highlights a clear potential for personalized medicine that would factor in microbiome composition.

However, we need to keep in mind the complexity of interactions among the gut microbiota. We must acknowledge the heterogeneity of microbial populations across individuals, and the potential consequences of this heterogeneity on treatment responses. Future studies should bridge knowledge gaps regarding specific microbial species involved in biotransformation of Polygonatum polysaccharides and their metabolites. Such studies could establish microbial population biomarkers for treatment responses to allow for personalization of treatment approaches based on individual microbiome profile.

Furthermore, as we advance our understanding of these interactions, we must also consider the ethical ramifications of manipulating gut microbiota for therapeutic purposes. Adverse effects, such as dysbiosis or unintended effects of microbial modulation, need to be considered carefully alongside the benefits of such interventions. Thorough clinical trials will need to be conducted to assess whether microbiota-targeted therapies are safe and effective and to ensure an appropriate balance between innovation and patient safety is maintained.

In summary, the investigation of gut microbiota in the bioconversion of Polygonatum polysaccharides and its effects on PD-L1 degradation represents an untapped opportunity in cancer treatment. This review encourages researchers to investigate the microbial universe to map complex relationships of importance to cancer therapeutics. Approaching this area of research with an interdisciplinary effort acts to unlock the maximal potential of gut microbiota and natural products in further development of cancer therapy. The future of cancer treatment may lie in the balance of our microbiome and the therapeutic agents we introduce.
